# Target Balloon-Assisted Antegrade and Retrograde Use of Re-Entry Catheters in Complex Chronic Total Occlusions

**DOI:** 10.3390/jcdd10020053

**Published:** 2023-01-29

**Authors:** Lorenzo Patrone, Nada Selva Theivacumar, Brahman Dharmarajah, Narayanan Thulasidasan, Athanasios Diamantopoulos, Luis Mariano Palena, Muliadi Antaredja, Lisa Tilemann, Erwin Blessing

**Affiliations:** 1 West London Vascular and Interventional Centre, London North West University Healthcare NHS Trust, Harrow HA1 3UJ, UK; 2 Section of Vascular Surgery, Department of Surgery & Cancer, Imperial College, London SW7 2AZ, UK; 3 Department of Interventional Radiology, Guy’s and St. Thomas’ NHS Foundation, Trust, London SE1 7EH, UK; 4 Endovascular Surgery Unit, Maria Cecilia Hospital—GVM Care & Research, 48033 Cotignola, Italy; 5 University Heart and Vascular Center, University Hospital Hamburg-Eppendorf, 20246 Hamburg, Germany

**Keywords:** peripheral arterial disease, chronic total occlusions, re-entry devices, SAFARI technique, retrograde recanalizations, target balloon-assisted recanalizations, level of evidence: level 3, non-randomized follow-up study

## Abstract

Purpose, Retrograde recanalizations have gained increasing recognition in complex arterial occlusive disease. Re-entry devices are a well described adjunct for antegrade recanalizations. We present our experience with target balloon-assisted antegrade and retrograde recanalizations using re-entry devices in challenging chronic total occlusions. Materials and Methods: We report data from a retrospective multicenter registry. Eligibility criteria included either antegrade or retrograde use of the Outback^TM^ or GoBack^TM^ re-entry catheter in combination with a balloon as a target to accomplish wire passage, when conventional antegrade and retrograde recanalization attempts had been unsuccessful. Procedural outcomes included technical success (defined as wire passage though the occlusion and delivery of adjunctive therapy with <30% residual stenosis at final angiogram), safety (periprocedural complications, e.g., bleeding, vessel injury, or occlusion of the artery at the re-entry site, and distal embolizations), and clinical outcome (amputation-free survival and freedom from target lesion revascularization after 12-months follow-up). Results: Thirty-six consecutive patients underwent target balloon-assisted recanalization attempts. Fourteen (39 %) patients had a history of open vascular surgery in the index limb. Fifteen patients were claudications (Rutherford Class 2 or 3, 21 presented with chronic limb threatening limb ischemia (Rutherford Class 4 to 6). The locations of the occlusive lesions were as follows: iliac arteries in 3 cases, femoropopliteal artery in 39 cases, and in below-the-knee arteries in 12 cases. In 15 cases, recanalization was attempted in multilevel occlusions. Retrograde access was attempted in 1 case in the common femoral artery, in the femoropopliteal segment in 10 cases, in below-the-knee arteries in 23 cases, and finally in 2 patients via the brachial artery. In 10 cases, the re-entry devices were inserted via the retrograde access site. Technical success was achieved in 34 (94 %) patients. There were 3 periprocedural complications, none directly related to the target balloon-assisted re-entry maneuver. Amputation-free survival was 87.8 % and freedom from clinically driven target lesion revascularization was 86.6 % after 12-months follow-up. Conclusion: Target balloon-assisted use of re-entry devices in chronic total occlusions provides an effective and safe endovascular adjunct, when conventional antegrade and retrograde recanalization attempts have failed.

## 1. Introduction

Over the past decade, there has been a paradigm shift in the treatment of infrainguinal arterial chronic total occlusions (CTOs). As endovascular technology, techniques and experience have developed, outcomes in amputation free survival and mortality have become equivalent to those of open surgery in the context of critical limb threatening ischemia [1].

Advances in endovascular techniques, operator’s skills as well as innovations in dedicated medical devices have helped to facilitate technical success rates and outcomes even in complex lesions. The Outback^TM^ re-entry catheter (Cordis Corporation, Bridgewater, NJ, USA) and the GoBack^TM^ device (Bentley InnoMed GmbH, Hechingen, Germany) are well described examples to aid recanalizations, helping in crossing back into the true lumen when conventional wire and catheter techniques have failed [2,3].

Successful wire passage through challenging occlusions was also improved through the introduction of retrograde recanalization techniques [4,5,6,7,8]. Especially in patients post endarterectomy of the common femoral artery, re-entering into the true lumen via the retrograde route without the use of a re-entry device can be very challenging or even impossible [9]. The combination of the Outback^TM^ re-entry catheter and retrograde vessel access, with the device deployed in a retrograde manner may be utilized in particularly complex chronic total occlusions [5,10]. In selected cases, a balloon positioned as a target for the needle of a re-entry device can further improve success rates. Although small cases series have been reported [11,12,13], there is little information regarding acute technical success rates and clinical follow-up in larger cohorts. We therefore present our multi-center experience of target balloon-assisted use of the Outback^TM^ and the GoBack^TM^ re-entry catheters in complex peripheral arterial revascularizations. 

## 2. Materials and Methods

### 2.1. Study Design

The study was conducted in accordance with the Declaration of Helsinki. The protocol was approved by the Institutional Review Boards. Due to the retrospective, non-interventional nature of the study based solely on data generated and documented during clinical practice, informed and written consent was not required in accordance with the statement of the Institutional Review Boards. A total of 36 consecutive patients were treated in 4 different institutions between October 2014 and February 2021. All cases were discussed by a multidisciplinary team prior to the intervention. Patients with claudication symptoms were offered endovascular therapy after failure of conservative and medical treatment and persisting lifestyle-limiting symptoms. Eligibility included all endovascular cases involving balloon-targeted use of the Outback^TM^ or the GoBack^TM^ re-entry catheter in procedures, where both antegrade and conventional retrograde recanalization were unsuccessful. 

Data were collated from the picture archiving and communication systems (PACS), radiology information systems (RIS) and electronic patient records in each institution. Patient and procedural characteristics were collected for gender, age, Rutherford clinical stage, history of previous vascular surgery in the treated limb, degree of calcification (PACCS score) [14], lesion complexity (TASC II classification) [15], pre- and post-interventional tibial run-off score, site of retrograde vessel access, target balloon re-entry site, and any subsequent procedural angioplasty or stenting. 

Technical success was defined as target balloon-assisted wire passage through the occlusion, delivery of adjunctive therapy and <30% residual stenosis at final angiogram. The procedural outcome measures included complications (distal embolization, bleeding, perforation etc.), amputation-free survival as well as freedom from clinically driven target lesion revascularization (cd-TLR) at 12-months follow-up.

### 2.2. Procedure

The decision to target the re-entry catheter to a balloon, entered either via the antegrade or retrograde route, was made at the primary operator’s discretion after failure of conventional recanalization attempts. Antegrade access was either from the ipsilateral common femoral artery, the brachial artery or the contralateral common femoral artery using a crossover technique. Five-thousand IU of heparine was administered after sheath placement. In longer procedure, ACT was measured with a target of >200 s.

**Figure 1:**
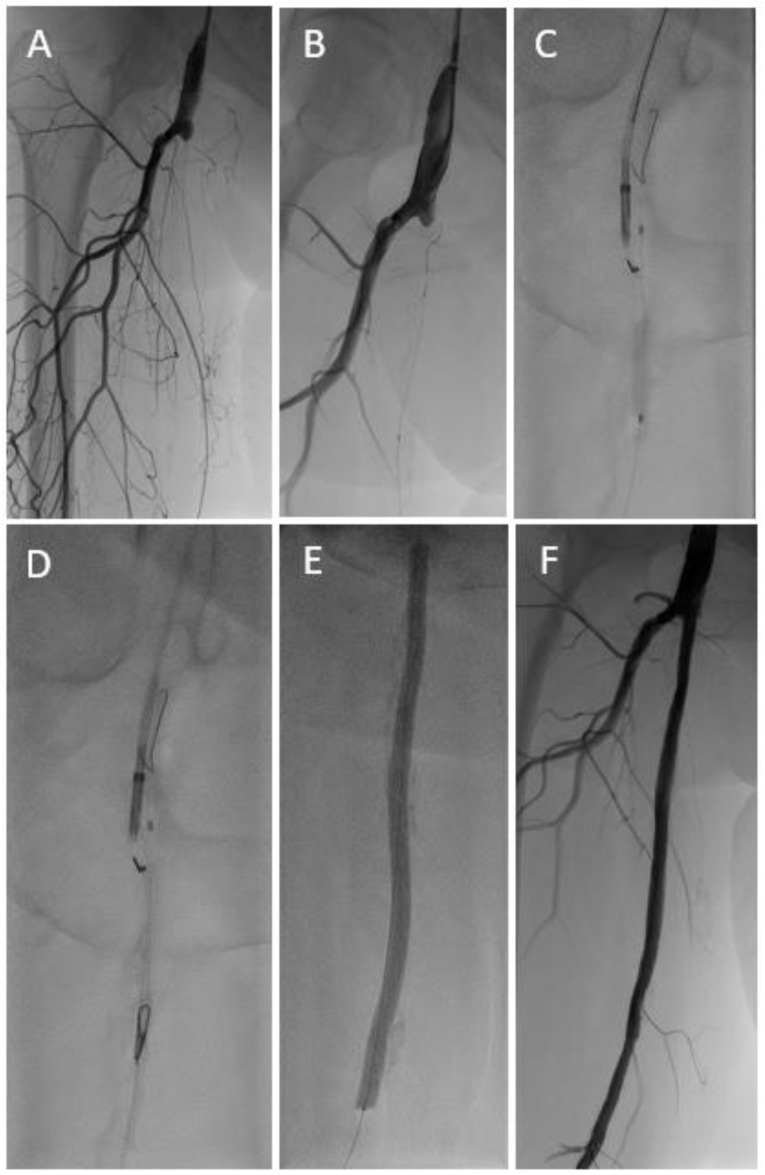
Long occlusive lesion (**A**) with initially unsuccessful recanalization attempts via both the antegrade and retrograde (**B**) route. A target-balloon was inserted via the retrograde access and punctured with the re-entry needle of an Outback™ catheter (**C**,**D**). After predilatation with a standard balloon followed by drug coated balloon angioplasty (**E**), an acceptable result was achieved without the need for subsequent stent implantation (**F**).

Distal retrograde access was accomplished with a 21-gauge micro-puncture access needle under either ultrasound or, in the majority of cases, using fluoroscopic guidance with an 0.018″ support wire, (V 18, 260 cm, Boston Scientific or Command, Abbott Medical) introduced. To prevent spasm, nitroglycerine was administered according to the discretion of the interventionalist. In cases of failed retrograde wire passage through the occlusion, the re-entry catheter was inserted either through a sheath as per Instructions for Use (IFU) or, in the interest of a low-profile approach, sheath-less, again at the primary operator’s discretion. In cases of retrogradely advanced target balloons, they were introduced either sheath-less or via a 4 F sheath. Target balloons (preferably Armada™ 18, Abbott Medical, range 3–5 mm, length 40–120 mm) were inflated with low pressure (2–4 atm). Re-entry into the target balloon was aimed at the most distal part of the balloon to allow maximal length of wire insertion (0.014″ Stabilizer™, Cordis or Glidewire Advantage™, Terumo) into the punctured balloon (Figure 1). In combination with the Outback device, in the majority of cases the burst balloon was then deflated and slowly removed under fluoroscopic guidance, with the wire coming from the opposite access advanced accordingly, in order to be kept into the burst lumen of the balloon. After wire externalization, adjunctive therapy of the occluded segment was performed via the antegrade route at the operator’s discretion. In cases where the re-entry catheter was used in a sheath-less fashion, after retrieval of the device, hemostasis was achieved via introduction of a 4 F sheath. Finally, closure of the retrograde access site was performed either using external manual compression, inflation of an external blood pressure cuff (100 mm Hg), or prolonged inflation (5 min) of a balloon at site of vessel access, advanced via the antegrade route. Protamine was not administered routinely.

Completion angiography at the treated segment as well as below-the-knee run-off was performed to assess treatment outcome and procedural complications. Postinterventional medical treatment varied between the centers. In general, in the absence of an indication for oral anticoagulant therapy, dual antiplatelet therapy for at least 4 weeks was most often recommended post-procedural, or more recently, low dose rivaroxaban in combination with single antiplatelet therapy.

### 2.3. Statistical Analysis

The number of observations represents patients. Continuous variables are reported as mean ± standard deviation (SD), median and inter-quartile range. Categorical data are presented as number and percentage. Freedom from clinically driven target lesion revascularization and amputation-free survival are reported with Kaplan–Meier methods. Statistical analysis was performed with SPSS software (version 25).

## 3. Results

### 3.1. Baseline Characteristics

**Figure 2:**
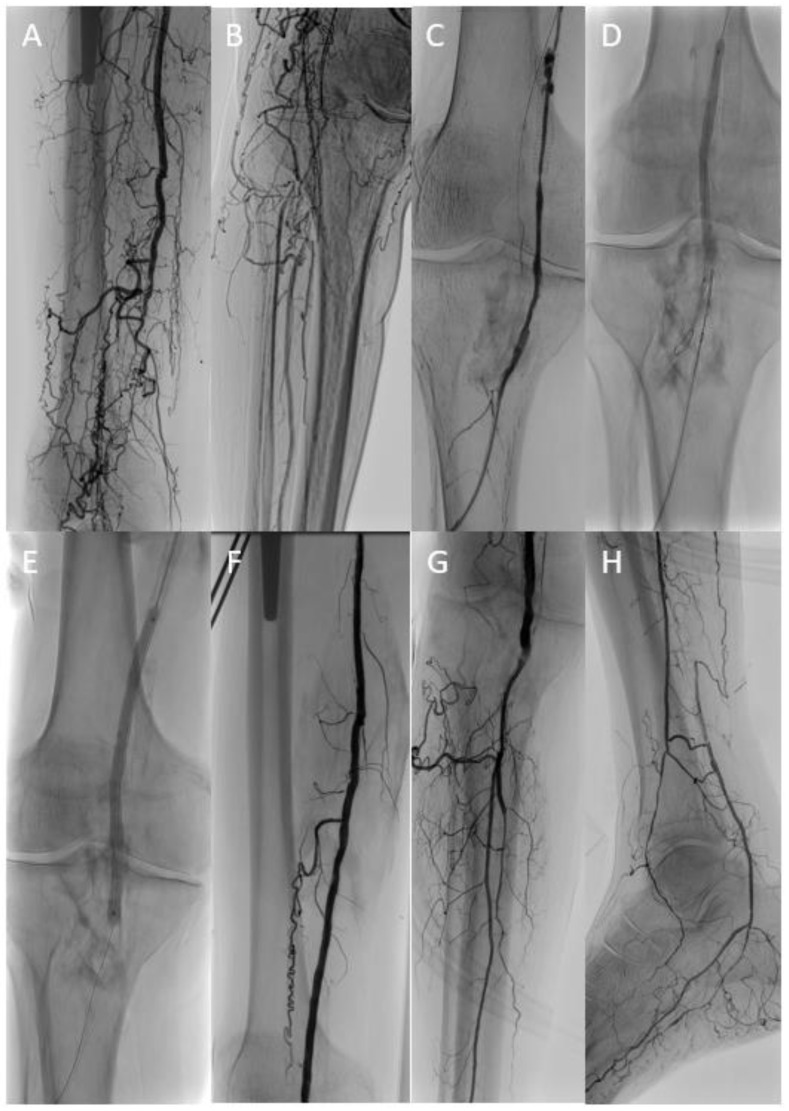
In a patient with critical limb ischemia and femoropopliteal occlusion (**A**,**B**), conventional recanalization attempts of the native artery and the chronically occluded femoropopliteal vein graft (**C**) were unsuccessful. Finally, target balloon-assisted re-entry with a retrogradely inserted GoBack™ catheter (**D**) enabled successful wire passage and adjunctive therapy of the native femoropopliteal segment (**E**–**G**) with a single-vessel run-off (**H**).

In 36 patients (mean age 75.3 ± 10.3; range 53 to 97 years), recanalization was attempted with target balloon-assisted re-entry using the Outback™ device (Figure 1) in 33 cases and the GoBack™ device (Figure 2) in 3 cases. Fifteen patients (42%) presented with claudication (Rutherford Stage 2 or 3), 1 patient had rest pain (Rutherford Stage 4), 17 patients (47%) digital ischemic ulcerations (Rutherford Stage 5), and 3 patients (8%) major gangrene (Rutherford Stage 6). Baseline patient and lesion characteristics are shown in Table 1 and Table 2.

### 3.2. Procedural Details

Fourteen patients (39%) had a prior history of open vascular surgery of the target limb, including common femoral endarterectomy, femoro-popliteal bypass, femoro-femoral bypass, and aorto-bifemoral bypass. In 10 patients (28%), the re-entry catheter was introduced via the retrograde route, in 20 cases (56%) retrograde access was established without the use of a sheath. Retrograde access arteries and balloon re-entry sites are shown in Table 2. All retrograde punctures were performed with patients in a supine position. 

### 3.3. Outcomes

Successful target balloon-assisted lesion recanalization could be achieved in 34 out of 36 patients (94%). In one patient with an occluded femoropopliteal bypass, despite additional retrograde access, the Outback™ re-entry catheter could not be positioned close enough to the target-balloon to allow the needle to reach the inflated balloon, most likely due to severe fibrosis at the area of the distal bypass anastomosis. The patient then underwent revascularization with a bypass and was discharged 10 days thereafter. In a second case, the Outback™ re-entry needle did not reach the balloon in an occluded common iliac artery. However, after wire escalation and alternative strategies (CART etc.), wire passage and adjunctive therapy could be accomplished. Since the initial strategy (target balloon-assisted re-entry) failed, we consider that case as not successful in respect to our registry.

In all remaining patients, wire passage via balloon-puncture and adjunctive therapy could be performed successfully with a documented patency and <30% residual stenosis at final angiogram. Recanalized segments and adjunctive therapy can be found in Table 2. 

**Figure 3:**
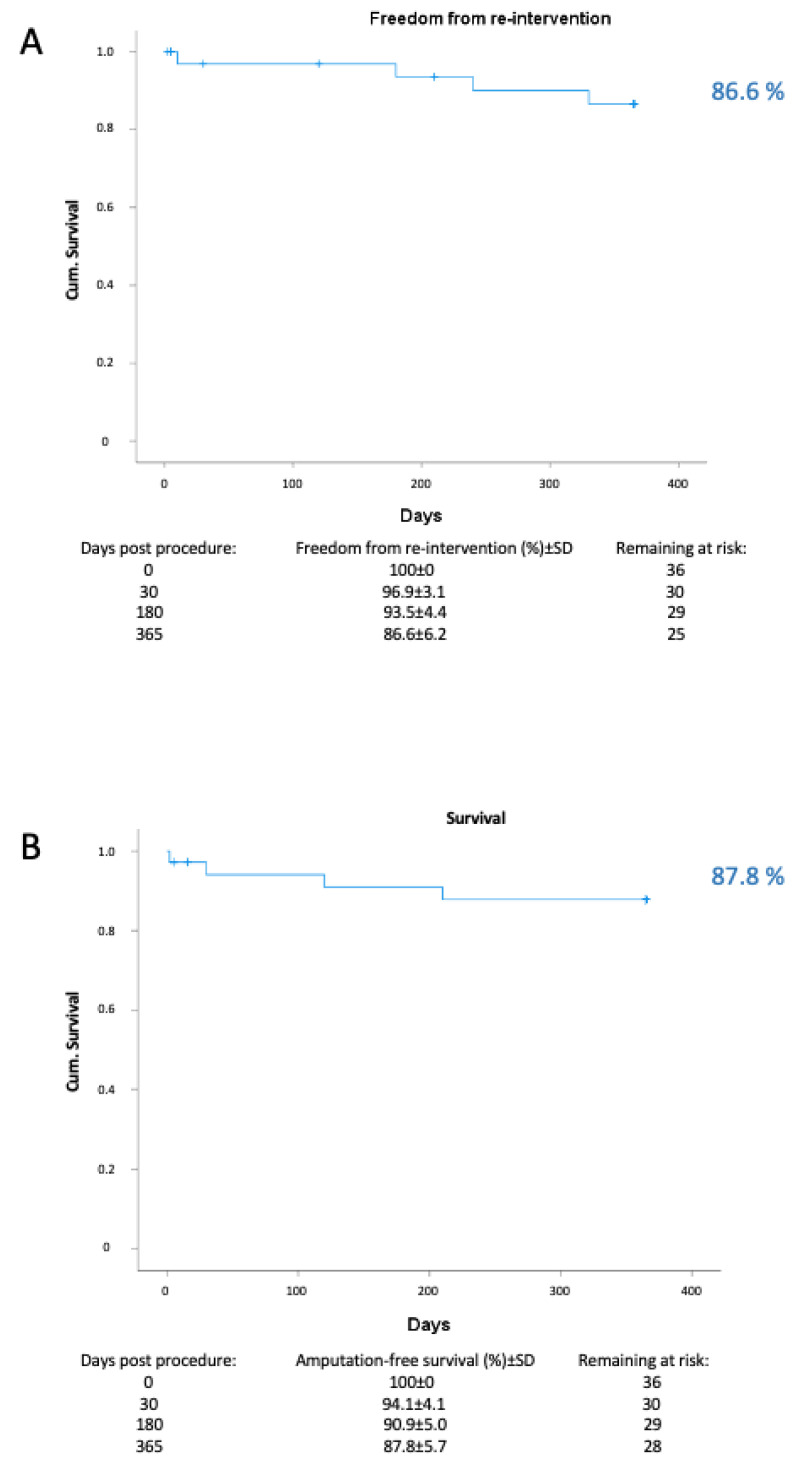
Kaplan–Meier analysis for (**A**) freedom from clinically driven target lesion revascularization (cd TLR) and (**B**) survival.

Four patients were lost to follow-up and 4 died within the first year. There were no major amputations. The survival rate was 87.8% and freedom from cd-TLR was 86.6% at 12 months (Table 3, Figure 3).

### 3.4. Complications

One patient with known coronary artery disease died 2 days after the intervention due to cardiac arrest. In one patient, perforation after aggressive lesion preparation in a severely calcified superficial femoral artery (PACCS score: 4) required implantation of a Viabahn™ endoprothesis. In another patient, 10 mg rt-PA were administered due to semi occlusive thrombus formation at the level of the tibioperoneal trunk. Although no bleeding was seen on the final angiogram, the patient experienced late bleeding complication 6 h after the procedure at the retrograde access site in the proximal anterior tibial artery. The patient developed compartment syndrome, requiring fasciotomy, with subsequent full recovery. According to SVS reporting standards [16], the cardiac arrest and the bleeding requiring fasciotomy were classified as severe, the perforation requiring implantation of an endoprothesis as moderate peri-interventional complications (Table 3). We did not observe any distal embolizations during the procedure (identical pre- and postinterventional distal run-off score). We also did not observe any formation of pseudoaneurysms or any other pathologic conditions at the site of re-entry during the follow up visits in any of our patients. In general, we strongly recommend to establish complication management procedures before starting a retrograde access program to manage bleeding (covered stents etc.) and distal embolizations (aspiration tools, etc.). As mentioned, there were no target limb major amputations at the 30-days and at 12-months follow up. 

## 4. Discussion

Our multicenter retrospective analysis demonstrated that target balloon-assisted use of re-entry devices is safe and effective and might help to further improve the technical success rates in challenging occlusions.

Subintimal recanalizations of occluded peripheral arteries were first described by Bolia et al. Technical success was achieved in 76% of cases in femoropopliteal occlusion with moderate lesion length [17]. After passage of the occluded segment via the subintimal space, re-entering into the true lumen can be challenging or even impossible, especially in long occlusions and severely calcified arteries. Dedicated re-entry devices are extremely helpful tools in those circumstances [2,10,18,19,20,21]. The use of re-entry catheters has been shown to avoid extending the length of dissection as well as the procedural time [2,22]. In addition, retrograde recanalizations are increasingly used in chronic total occlusion. The so-called “subintimal arterial flossing with antegrade-retrograde intervention” (SAFARI)-technique was first described by Spinosa et al. [4]. In their rather small cohort of patients with critical limb ischemia and failed antegrade attempt, a combination of antegrade and retrograde subintimal recanalization allowed successful wire passage and adjunctive therapy in all cases. Retrograde recanalization resulted in lower dissection severity and lower rates of consecutive stent placement as compared to extended antegrade attempts in another relatively small study [7]. A recent large single center cohort study demonstrated high technical success and low complication rates with retrograde tibioperoneal access in 554 patients with infrainguinal occlusions [6]. Growing confidence in retrograde access encourages experienced interventionalists to use this approach not only in patients with critical limb threatening ischemia but also increasingly in patients affected by lifestyle-limiting claudication [6]. Under certain circumstances the combination of retrograde access and the use of dedicated re-entry devices can also help to further increase acute the technical success rates. Retrograde insertion of re-entry devices was shown to be safe and effective in challenging infrainguinal occlusions [5,9,10]. The use of a catheter inserted from the retrograde approach, to guide the re-entry, was described in a case report by Bozlar et al. in 2008 [23]. Subsequently, a balloon positioned as a target for the re-entry device at the site of intended wire passage, introduced either via the antegrade or retrograde route, was described to facilitate recanalization of occluded segments [11,12,13]. Tai et al. reported a small case series with target balloon-assisted recanalizations with both the Outback™ and the Pioneer Plus™ catheter in patients with critical limb ischemia [13]. The Pioneer Plus^TM^ catheter incorporates intravascular ultrasound imaging to potentially further enhance the precision and safety of the re-entry but also adding costs to the procedure. In another report, target balloon-assisted use of the Outback™ enabled recanalization of a thrombosed femoropopliteal bypass graft [11]. In all cases, the re-entry device was advanced from antegrade to puncture into a balloon, which was advanced via the retrograde route. In contrast, in our study, in 10 out of the 36 cases, the re-entry device introduced from retrograde was an increasing preference for a sheath-less application. Sheath-less insertion of the Outback™ catheter enables an outer diameter of 5.9 F rather than the 8 F outer diameter of a 6 F sheath recommended as per the IFU. With this reduction in diameter, trauma and hemostasis at the access sites was not a relevant issue in our study. The rather recently launched GoBack™ device can be introduced via a 4 F sheath or possibly also be used in a sheath-less manner. The retraction of the burst balloon containing the wire inserted within it from the opposite access is the most delicate step of this technique and requires two operators, in order not to inadvertently loose the connection between the wire and the balloon. A low threshold to adoption of this approach may also be beneficial in saving procedural time, contrast medium usage, and radiation dose to both the patient and the operator. 

This study is limited by its retrospective design and data acquisition. Therefore, we cannot report on the important clinical outcome parameters (e.g., change in Rutherford class, quality of life, duplex scan, etc.) in our cohort. It has been performed across four international centers with multiple operators with different decision making processes, non-standardized post-interventional treatment protocol, etc. There was also no comparator group in this study, but as described, this intervention was only reserved as a “bail-out“-option after conventional revascularization attempts had failed. Of note, there is a certain learning curve regarding the use of re-entry devices, and they add a significant cost to the procedure. A significant number of patients were treated despite presenting with claudication instead of critical limb threatening ischemia. Our approach could be considered rather aggressive and is not necessarily in accordance with current guidelines. However, we strongly believe that, where conventional revascularization methods have failed, the low complication rate demonstrated by our multicenter operators, justifies endovascular treatment, including target balloon-assisted retrograde recanalizations in patients presenting with lifestyle-limiting claudication as well as critical limb threatening ischaemia.

## 5. Conclusions

In summary, target balloon-assisted retrograde application of the Outback™ and GoBack™ re-entry catheter is feasible and safe as demonstrated in our retrospective multi-center cohort study. It provides a valuable option in CTOs, where both antegrade and conventional retrograde subintimal recanalization attempts have failed. 

## Figures and Tables

**Table 1:** Patient characteristics (n = 36).

Age	75.3 ± 10.3
Sex	male: 29 (81 %); female: 7 (19 %)
Diabetes mellitus	18 (50 %)
Hyperlipidemia	20 (56 %)
Smoking (former or current)	14 (39 %)
Hypertension	34 (94 %)
Chronic kidney disease *	9 (25 %)
Rutherford class	4.2 ± 1.1 2: 1 (3 %) 3: 14 (39 %) 4: 1 (3 %) 5: 17 (47 %) 6: 3 (8 %)

* GFR < 50 mL/min. Continuous data are presented as mean ± SD. Categorical data are presented as %.

**Table 2:** Lesion and procedural characteristics (n = 36).

TASC: B C D	5 (14%) 12 (33%) 19 (53%)
Lesion length (cm)	27 ± 13 (25; 1–53)
Length occlusion (cm)	23 ± 13 (21; 1–53)
PACSS Calcium Score: 1 2 3 4	5 (14%) 12 (33%) 4 (11%) 15 (42%)
Re-entry device: Outback™ GoBack™	33 3
Balloon re-entry site: Iliac arteries CFA SFA Popliteal artery Below-the-knee	3 (8 %) 2 (6 %) 16 (44 %) 9 (25 %) 6 (17 %)
Recanalized artery: Iliac arteries SFA Popliteal artery Below-the-knee Multilevel	3 (8 %) 21 (58 %) 18 (50 %) 12 (33 %) 15 (42 %)
Pre-interventional run-off below-the-knee	1.7 ± 1.8 (2; 0–3)
Post-interventional run-off below-the-knee	1.7 ± 1.8 (2; 0–3)
Previous surgery index limb	14 (39 %)
Total procedure time (min)	134 ± 68 (116; 59–360)
Fluoroscopy time (min)	48 ± 21 (47; 16–118)
Total radiation dose (cGy x cm^2^)	4059 ± 2669 (3210; 1203–14,221)
Total contrast (mL)	156 ± 77 (160; 9–450)
Re-entry device inserted via retrograde access	10 (28 %)
Re-entry device inserted sheath-less	5 (14 %)

PACSS: peripheral arterial calcium scoring system; CFA: common femoral artery; SFA: superficial femoral artery; Continuous data are presented as mean ± SD, median and inter-quartile range. Categorical data are presented as %.

**Table 3:** Main study outcomes (n = 36).

Technical Success *	94% (34/36)
Complications: Mild Moderate ^§^ Severe	0% (0/36) 5% (2/36) 0% (0/36)
30-days follow-up: Lost for follow-up Target limb major amputation Amputation free survival Freedom from cd-TLR ^‡^	14% (5/36) 0% (0/36) 97% (30/31) 100% (29/29)
12-months follow-up: Lost for follow-up Target limb major amputation Amputation free survival Freedom from cd-TLR ^‡^	25% (9/36) 0% (0/36) 92% (23/25) 83% (20/24)

* defined as successful wire passage and <30% residual stenosis at final angiogram; § perforation after balloon inflation, successfully treated with implantation of a covered stent. In another patient, bleeding at site of retrograde access 6 h after lysis, resulting in compartment syndrome, successfully treated with fasciotomy, patient recovered fully; ‡ referring to the cohort of successful recanalizations with available follow-up; cd-TLR: clinically driven target lesion revascularization.

## Data Availability

The underlying datasets generated and/or analyzed during the current study are available from the corresponding author upon reasonable request.

## References

[1] Jones W.S., Dolor R.J., Hasselblad V., Vemulapalli S., Subherwal S., Schmit K., Heidenfelder B., Patel M.R. (2014). Comparative effectiveness of endovascular and surgical revascularization for patients with peripheral artery disease and critical limb ischemia: Systematic review of revascularization in critical limb ischemia. Am. Heart J..

[2] Kitrou P., Parthipun A., Diamantopoulos A., Paraskevopoulos I., Karunanithy N., Katsanos K. (2015). Targeted True Lumen Re-Entry With the Outback Catheter: Accuracy, Success, and Complications in 100 Peripheral Chronic Total Occlusions and Systematic Review of the Literature. J. Endovasc. Ther..

[3] Rodriguez L.E., Tabrizi R., Malgor R.D., Wohlauer M., Jacobs D.L. (2021). Sharp Recanalization with the Upstream GoBack Catheter for Chronic Occlusive Ilio-Caval Thrombosis. Ann. Vasc. Surg..

[4] Spinosa D.J., Harthun N.L., Bissonette E.A., Cage D., Leung D.A., Angle J.F., Hagspiel K.D., Kern J.A., Crosby I., Wellons H.A. (2005). Subintimal arterial flossing with antegrade-retrograde intervention (SAFARI) for subintimal recanalization to treat chronic critical limb ischemia. J. Vasc. Interv. Radiol..

[5] Schmidt A., Bausback Y., Piorkowski M., Werner M., Bräunlich S., Ulrich M., Varcoe R., Friedenberger J., Schuster J., Botsios S. (2012). Retrograde recanalization technique for use after failed antegrade angioplasty in chronic femoral artery occlusions. J. Endovasc. Ther..

[6] Schmidt A., Bausback Y., Piorkowski M., Wittig T., Banning-Eichenseer U., Thiele H., Aldmour S., Branzan D., Scheinert D., Steiner S. (2019). Retrograde Tibioperoneal Access for Complex Infrainguinal Occlusions: Short- and Long-Term Outcomes of 554 Endovascular Interventions. JACC Cardiovasc. Interv..

[7] Giusca S., Lichtenberg M., Hagstotz S., Eisenbach C., Katus H.A., Erbel C., Korosoglou G. (2020). Comparison of ante-versus retrograde access for the endovascular treatment of long and calcified, de novo femoropopliteal occlusive lesions. Heart Vessels.

[8] Giannopoulos S., Palena L.M., Armstrong E.J. (2021). Technical Success and Complication Rates of Retrograde Arterial Access for Endovascular Therapy for Critical Limb Ischaemia: A Systematic Review and Meta-Analysis. Eur. J. Vasc. Endovasc. Surg..

[9] Patrone L., Dharmarajah B., Korosoglou G., Theivacumar S., Antaredja M., Oberacker R., Tilemann L., Blessing E. (2022). Retrograde use of the Outback re-entry catheter in complex infrainguinal arterial recanalizations. J. Vasc. Surg..

[10] Patrone L., Stehno O. (2019). Retrograde insertion of the outback reentry device from a tibial artery for complex infrainguinal recanalization. CVIR Endovasc..

[11] Kwak J.C., Chung H., Lee S., Yeom S., Cha S. (2015). Target balloon-assisted antegrade and retrograde approach for recanalization of thrombosed fem-pop bypass graft using the outback catheter. J. Korean Soc. Radiol..

[12] Goltz J.P., Anton S., Wiedner M., Barkhausen J., Stahlberg E. (2017). Simultaneous Antegrade-Retrograde Subintimal Revascularization of a Femoropopliteal Chronic Total Occlusion by a Reentry Device-Facilitated Puncture of a Retrogradely Inserted Balloon. J. Endovasc. Ther..

[13] Tai Z., Lee A. (2015). Reentry-catheter assisted SAFARI technique. J. Invasive Cardiol..

[14] Rocha-Singh K.J., Zeller T., Jaff M.R. (2014). Peripheral arterial calcification: Prevalence, mechanism, detection, and clinical implications. Catheter. Cardiovasc. Interv..

[15] Norgren L., Hiatt W.R., Dormandy J.A., Nehler M.R., Harris K.A., Fowkes F.G. (2007). Inter-Society Consensus for the Management of Peripheral Arterial Disease (TASC II). J. Vasc. Surg..

[16] Stoner M.C., Calligaro K.D., Chaer R.A., Dietzek A.M., Farber A., Guzman R.J., Hamdan A.D., Landry G.J., Yamaguchi D.J. (2016). Reporting standards of the Society for Vascular Surgery for endovascular treatment of chronic lower extremity peripheral artery disease: Executive summary. J. Vasc. Surg..

[17] Bolia A., Miles K.A., Brennan J., Bell P.R. (1990). Percutaneous transluminal angioplasty of occlusions of the femoral and popliteal arteries by subintimal dissection. Cardiovasc. Interv. Radiol..

[18] Schmidt A., Keirse K., Blessing E., Langhoff R., Diaz-Cartelle J. (2014). Offroad re-entry catheter system for subintimal recanalization of chronic total occlusions in femoropopliteal arteries: Primary safety and effectiveness results of the re-route trial. J. Cardiovasc. Surg. (Torino).

[19] Wosik J., Shorrock D., Christopoulos G., Kotsia A., Rangan B.V., Roesle M., Maragkoydakis S., Abdullah S.M., Banerjee S., Brilakis E.S. (2015). Systematic Review of the BridgePoint System for Crossing Coronary and Peripheral Chronic Total Occlusions. J. Invasive Cardiol..

[20] Scheinert D., Bräunlich S., Scheinert S., Ulrich M., Biamino G., Schmidt A. (2005). Initial clinical experience with an IVUS-guided transmembrane puncture device to facilitate recanalization of total femoral artery occlusions. EuroIntervention.

[21] Gandini R., Fabiano S., Spano S., Volpi T., Morosetti D., Chiaravalloti A., Nano G., Simonetti G. (2013). Randomized control study of the outback LTD reentry catheter versus manual reentry for the treatment of chronic total occlusions in the superficial femoral artery. Catheter. Cardiovasc. Interv..

[22] Kawasaki D., Fukunaga M., Nakata T., Kato M., Ohkubo N. (2017). Comparison of the OUTBACK(®) Elite Reentry Catheter and the Bi-directional Approach after Failed Antegrade Approach for Femoro-popliteal Occlusive Disease. J. Atheroscler. Thromb..

[23] Bozlar U., Shih M.C., Harthun N.L., Hagspiel K.D. (2008). Outback catheter-assisted simultaneous antegrade and retrograde access for subintimal recanalization of peripheral arterial occlusion. Clin. Imaging.

